# Auditory interaction between runners: Does footstep sound affect step frequency of neighboring runners?

**DOI:** 10.1371/journal.pone.0280147

**Published:** 2023-01-06

**Authors:** Hiroaki Furukawa, Kazutoshi Kudo, Kota Kubo, Jingwei Ding, Atsushi Saito

**Affiliations:** 1 Department of Life Sciences, Graduate School of Arts and Sciences, The University of Tokyo, Tokyo, Japan; 2 Graduate School of Interdisciplinary Information Studies, The University of Tokyo, Tokyo, Japan; 3 Faculty of Occupational Therapy, Department of Rehabilitation, Kyushu Nutrition Welfare University, Kitakyushu, Fukuoka, Japan; 4 Graduate School of Human-Environment Studies, Kyushu University, Fukuoka, Japan; 5 Faculty of Human-Environment Studies, Kyushu University, Fukuoka, Japan; Fondazione Santa Lucia Istituto di Ricovero e Cura a Carattere Scientifico, ITALY

## Abstract

This study aimed to investigate the effect of footsteps of a neighboring runner (NR) on the main runner’s step frequency (SF), heart rate (HR), and rating of perceived exertion (RPE). The participants were male long-distance runners belonging to a university track and field team. Two experiments were conducted in which the main runner (participant) and NR (examiner) ran with the same running speed on two adjacent treadmills separated by a thin wall. The participants were instructed that the experimental purpose was to investigate the HR when running with others and running alone. In Experiment 1, NR performed three trials of changing the footstep tempo in 5 bpm (beat per minute) faster (+5bpmFS), 5 bpm slower (-5bpmFS), or no footsteps (NF) conditions. The results showed that the footstep condition affected the variability of the SF but not the mean SF. Next, Experiment 2 was conducted by increasing the footstep tempo condition. NR performed seven trials of changing the footstep tempo by ±3 bpm, ±5 bpm, ±10 bpm, or no footstep. The results showed that the footstep condition affected the mean SF and the SF decreased at -10bpmFS compared to NF. There were no differences in the HR and RPE between conditions. These results indicated that the footsteps of NR could influence the SF, although it was unclear whether footsteps were involved in the synchronization between runners. Overall, our findings emphasize the environmental factors that influence running behavior, including the NR’s footsteps.

## Introduction

In running competitions, there are two types of situations: running alone and running with people. In the 60m, 1500m and 3000m time trials, studies show that the performance can be better in head-to-head than when running alone [1–3]. It is not clear why running with others improves performance in long-distance running, or how the differences in the conditions affect performance. Previous studies have reported the following factors: drafting (reduction of aerodynamic drag by the preceding runner) [4–6], improvement of arousal level by social facilitation [7–9], and changes in attentional focus [10–12].

Each runner has a unique step frequency (SF) (i.e. the number of steps per minute) that optimizes their performance [13, 14]. However, the SF of two neighboring runners (NR) may leave their unique range and intermittently get close, which is considered to be a "synchronization" between runners [13]. In the 100m final of the 2009 World Championships in Athletics in Berlin, Usain Bolt set a world record, and Tyson Gay, who came in second, set the world’s second-best record. Analysis of SF of these two runners revealed that although their unique SFs were different in the semifinals, they were intermittently close in the finals, suggesting the possibility of synchronization [13]. However, it is not clear what kind of visual or auditory information causes this phenomenon. Moreover, no previous study has demonstrated that synchronization has a positive effect on running performance. As it occurs even in top athletes with optimized running movements, it is desirable to study the relationship between synchronization and performance and how it occurs [13].

It has been widely shown that auditory information entrains movement tempo (i.e. the number of beats or steps per minute; e.g., see Refs. 15–20), and this phenomenon is called the "entrainment" of movement tempo by auditory information [15, 16]. Auditory information with a certain tempo can also entrain SF of walking and running movements and that the tempo of SF approaches that of auditory information [16–20]. "Music” has elements of melody and harmony along with tempo and also simple beat sounds (e.g., metronome) which entrain SF of walkers and runners [19, 20]. A characteristic auditory information during running with others is the footsteps of others, which may cause SF entrainment.

A meta-analysis of the effects of music on the feeling scale, heart rate (HR), oxygen consumption (VO_2_), rating of perceived exertion (RPE), and performance has shown that listening to music during exercise improves the feeling scale, VO_2_, RPE, and performance [21]. Specifically, synchronizing the tempo of auditory information with SF reduces physiological load and produces better performance [22, 23]. Even for simple beats without melody and harmony, SF-synchronized beats can improve performance [23]. This positive effect has been attributed to the improvement in contractile efficiency of active muscles and the reduction in metabolic cost due to synchronization with auditory stimulation [22, 24, 25].

When running with another runner, the footsteps of the other runner can be considered as external auditory information, and when SF synchronization occurs between the two runners [13], the footsteps of the other runner approach a state in which the auditory stimuli are synchronized with SF. Similar to a metronome or music, it may affect SF synchronization, physiological load, and performance.

In an experiment in which two participants walked side-by-side while their visual information was cut off, their SFs synchronized even when they were not instructed to do so [26–28]. Hence, the sound of each other’s footsteps (auditory information) may affect SF, resulting in unintentional synchronization. However, it has not been clarified whether the footsteps of other runners affect SF during running, and no study shows a relationship between unintentional synchronization between two runners and their footsteps. Moreover, the effects of the footsteps of other runners on physiological load, RPE, and performance have not been sufficiently examined.

Therefore, the study aims to investigate (1) whether the different footstep tempo of the NR affect the SF of the main runner, and (2) the effects of NR footsteps on HR and RPE of the main runner.

In Experiment 1, we hypothesized that the footstep tempo of an NR would cause entrainment of the main runner’s SF, and examined its effect on SF. We set up an experimental situation in which the footsteps of a NR running side-by-side were manipulated based on the SF of the main runner to study the effect of the footsteps of one runner on the other. In Experiment 2, larger number of tempo conditions were adopted than those in Experiment 1 in order to examine the effect of the wider range of footstep tempo on the SF.

## Methods

### Participants

Healthy male trained distance runners participated in Experiment 1 (N = 10) and Experiment 2 (N = 16). In Experiment 2, one participant was excluded from this analysis because he had a within-participant standard deviation (SD) of SF greater than 3 SDs from the mean across participants. Therefore, we adopted the data for 15 participants. The mean ages (±SD) were 20.7±1.4 years and 20.9±1.6 years, the mean height was 169.4±4.8 cm and 170.4±3.8 cm, the mean body mass was 56.5±3.6 kg and 56.1±4.2 kg, the best time for a 5000-m in the current year was 16 min 22s ± 1 min 1 s and 16 min 6 s±44 s, in Experiments 1 and 2, respectively. They had practiced distance running on a university track and field team, and had trained for at least 40 minutes per day, four days a week for the past month. Four out of ten participants in Experiment 1 also participated in Experiment 2. Before the experiment, we explained the outline and possible risks in writing and orally to the participants, and obtained their consent to participate. This study was approved by the Ethics Committee of the Department of Health and Sports Science, Graduate School of Human and Environmental Studies, Kyushu University.

### Experiment 1

#### Experimental procedure and setup

After arriving at the laboratory, the participants’ blood pressure, resting HR, and body mass were recorded, and the experimental procedures were explained. The participants were instructed that the purpose was to conduct "an experiment to investigate the HR when running alone and with two people," but the original purpose was not revealed. After stretching, an HR measurement sensor (WearLink+ Coded Transmitter 31 XS-S, Polar) was attached to the chest, and an acceleration sensor (Stride Sensor WIND, Polar) was attached to the right shoe’s laces. Using these devices, HR and SF data were obtained every 5 s as beats per minute (bpm) and rotations per minute (rpm). A 5-min warm-up run was performed on one of the two adjacent treadmills (right side). The first trial of the experiment was started after a 5-min break. The running speed in the warm-up run and the main experiment was equivalent to approximately 70% HRmax. Based on the American Heart Association, the maximum HR estimated as “220-age” was used [29].

#### Stimuli

**Fig 1:**
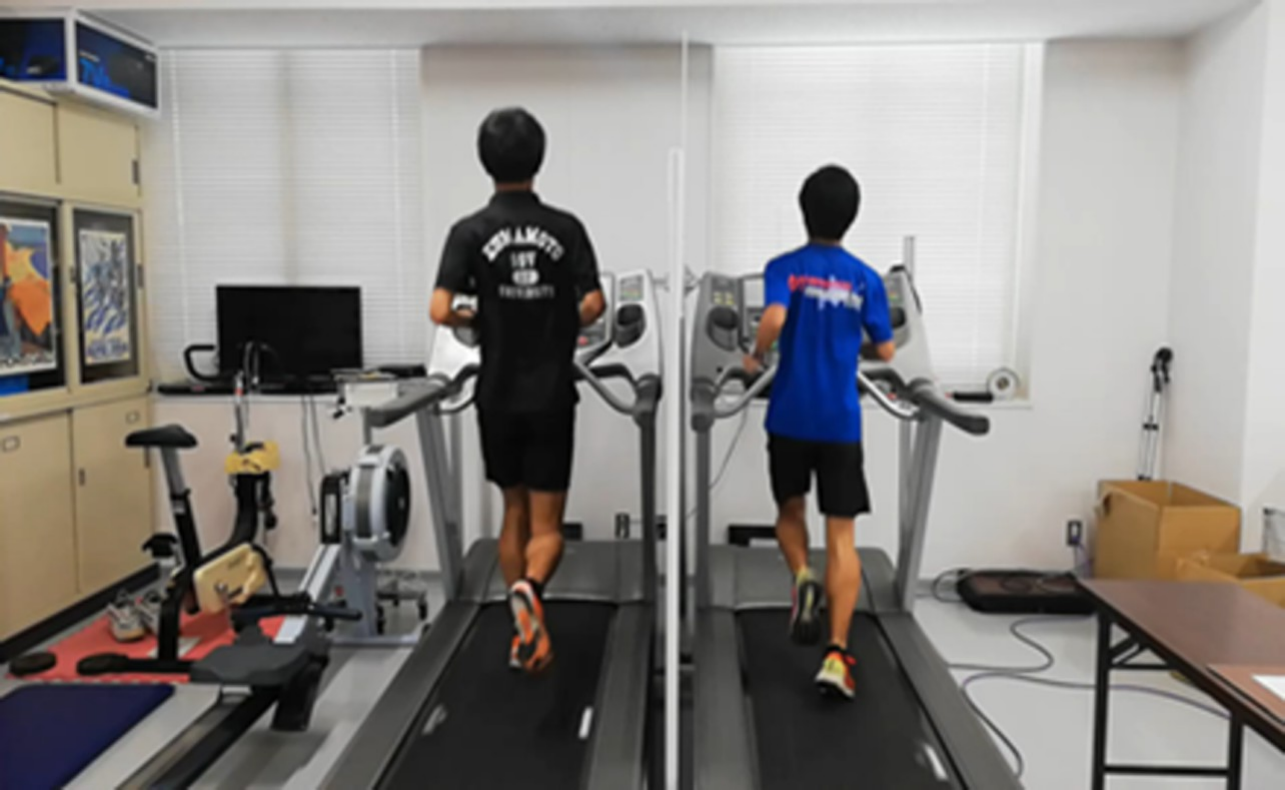
Experimental situation.

A thin wall (200 cm long, 170 cm wide, 6 cm thick, white) was placed between the two treadmills so that the runners could not see each other and only hear the footsteps (Fig 1). The running time per trial was 7 min and 30 s, and a constant speed was set, resulting in approximately 70% of HRmax (hereinafter “setting speed”). Totally, three trials were performed at the same setting speed. The following three conditions (1) to (3) were randomized and performed one at a time in a counterbalanced order. The rest period between trials was five minutes. Referring to the study by Dyck et al. [16], which showed that the tempo of music approached the running SF, the change rate in the footstep tempo of NR was set at ±5 bpm (equivalent to approximately ±3%).

#### Conditions

1. Footsteps +5 bpm condition (+5bpmFS)
  The participant ran at a setting speed for 7 min and 30 s, while the NS ran at the same speed for the same time, manipulating his steps. In the first 5 min, the NS listened to a metronome beeping at the same tempo as the participant’s cadence (i.e. the number of ipsilateral steps per minute) and synchronized with the sound. Therefore, the participant listened to both his footsteps and that of the NR, which were generated at a tempo approximated to their SF. The NR increased the cadence by 2.5 rpm from 5 min after the start of each trial, based on the participant’s average cadence from 4 min 40 s to 5 min ("reference time 1"). Five minutes after the start of each trial, the NR increased the cadence by 2.5 rpm. This operation increased SF (the number of steps per min) by 5 steps per minute (spm), and the participant heard footsteps 5 bpm faster. The change in the cadence of NR was performed in 5 s. For example, if the participant was running at a cadence of 90 rpm, 5 min after the start of the run, the cadence of the NR was increased to 92.5 rpm over 5 s. The participant heard footsteps with a tempo of 185 spm (92.5 rpm or 185 bpm) after 5 min and 5 s. To adjust the tempo of the metronome sound, the cadence of the participant measured by the accelerometer was displayed on a running computer (RS800CX, Polar), and the tempo of the metronome was adjusted to this value.
2. Footsteps -5 bpm condition (-5bpmFS)
  As in the +5bpmSF condition, the participant ran at a setting speed for 7 min and 30 s, while the NR ran at the same speed simultaneously and manipulated his steps. The NR’s cadence was set to twice that of the participant’s mean at the reference time 1, and his SF was slowed down by 5 spm from 5 min after the start of each trial. The rest of the procedure was the same as described in (1).
3. No footstep condition (NF).
  The NR walked silently from the start to the end of the run. The participant ran at a constant running speed for 7 min and 30 s.

### Measurements

#### Cadence

The cadence was measured every 5 s using an accelerometer attached to the right shoe’s laces and recorded on a running computer (RS800CX, Polar). The cadence at reference time 1 was defined as the "reference cadence," and after reference time 1 was defined as the "post-change cadence." To confirm the degree of increase or decrease in cadence due to the change in tempo of the participants’ footsteps, the ratio of the change to the mean reference cadence was calculated as the "SF change rate" using the following formula:

$$SF\;change\;rate\;(\%)=\frac{Post-change\;cadence-ave(Reference\;cadence)}{ave(Reference\;cadence)}\times 100$$

Data from 5 min to 5 min and 10 s as well as from the last 20 s (7 min and 10 s to 7 min and 30 s) were not included in the analysis because the participant could change the running motion at the end of the run [16].

#### HR

The HR was measured every 5 s by a chest-mounted HR sensor and recorded on a running computer (RS800CX, Polar). The mean HR from the beginning to 5 min after the start of each trial and that from 5 min after the start to the end were analyzed. As with cadence, data from 5 min to 5 min and 10 s after the start of each trial and data from the last 20 s (7 min and 10 s to 7 min and 30 s) were not included in the analysis.

#### RPE

The Japanese version of the Borg scale developed by Onodera et al. [30] was used. The participant verbally reported the RPE displayed on the panel at 1, 3, and 5 min after the start of each trial.

### Data analysis

Statistical data analysis was conducted using IBM SPSS Statistics version 25.0 (IBM Corp). Two-way repeated-measures analysis of variance (ANOVA) was used for the SF change rate, HR, and RPE, with factors of the footstep condition (i.e., +5bpmFS, -5bpmFS, and NF) and time. One-way repeated-measures ANOVA was used for the SD of SF. Bonferroni-adjusted multiple comparison tests were used to assess pairwise comparisons when significant main or interaction effects were found. Greenhouse-Geisser corrections were applied where sphericity was violated. The significance level and the marginally significant level were set at 5% and 10%, respectively. All results are presented as mean ± SD.

### Experiment 2

#### Experimental procedure and conditions

The same procedure as in Experiment 1 was used along with the +3 bpm footsteps (+3bpmFS), - 3 bpm footsteps (-3bpmFS), + 10 bpm footsteps (+10bpmFS), and—10 bpm footsteps (-10bpmFS) conditions. To confirm the reproducibility of the results of Experiment 1, +5bpmFS, -5bpmFS, and NF were also performed. The running time per trial was shortened from 7 min and 30 s to 5 min and 30 s to reduce the effect of fatigue due to the increase in the total running time [15]. The running speed was also set to the speed at which the participant could continue running comfortably for 60 min, and was determined by self-selection by adjusting the speed of the treadmill during the warm-up run. Moreover, we used devices (ForeAthlete245, GARMIN and Running Dynamics Pod, GARMIN) that can measure SF (spm) instead of cadence (rpm) to measure SF changes in more detail. Using these devices, SF data was obtained every 10 s in spm.

### Data analysis

Two-way repeated-measures ANOVA was used for the SF change rate, HR, and RPE. Bonferroni-adjusted multiple comparison tests assessed pairwise comparisons when significant main or interaction effects were found. One-way repeated-measures ANOVA was used for the SD of SF. Bonferonni-adjusted multiple comparison tests assessed pairwise comparisons when significant main or interaction effects were found. Greenhouse-Geisser corrections were applied where sphericity was violated. The significance level was set at 5%.

## Results of Experiment 1

### SF change rate

**Fig 2:**
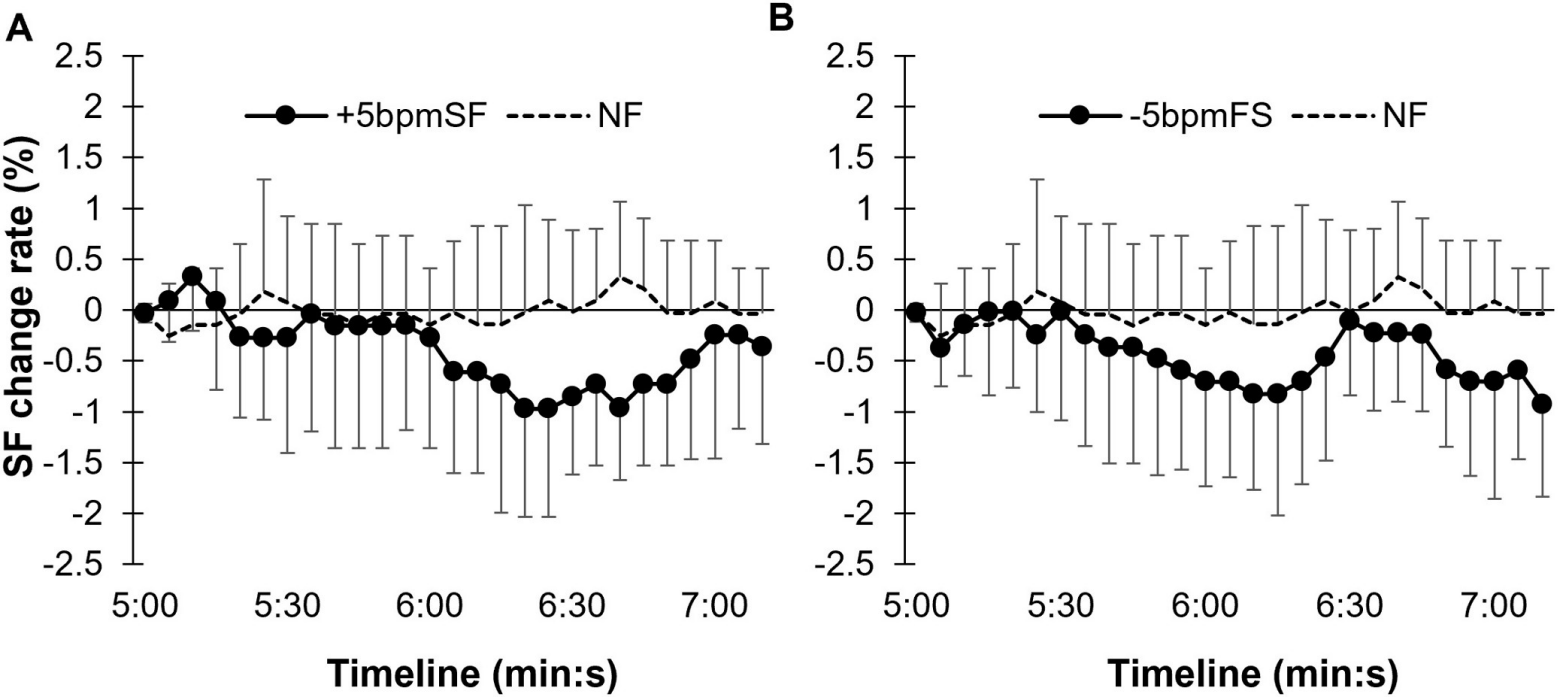
Changes in the SF change rate every 5 s in Experiment 1. The error bars represent between-participant SD. Compared to no footsteps condition (NF), the step frequency seems to decrease in the +5 bpm (A) and -5 bpm (B) footsteps condition but there were no significant differences between these conditions.

Fig 2 shows the SF change rate every 5 s in the +5bpmFS (A), -5bpmFS (B), and NF conditions. A two-way (condition × time) ANOVA showed that there was no interaction (F[5.05, 45.47] = 1.44, *p* = 0.23, $\eta_{p}^{2}$ = 0.14). There was no significant main effect of the condition on SF change rate (F[2, 18] = 0.80, *p* = 0.47, $\eta_{p}^{2}$ = 0.082). However, there was the marginal main effect of time on SF change rate (F[4.51, 40.62] = 2.41, *p* = 0.058, $\eta_{p}^{2}$ = 0.21). Multiple comparison showed no differences between conditions.

**Fig 3:**
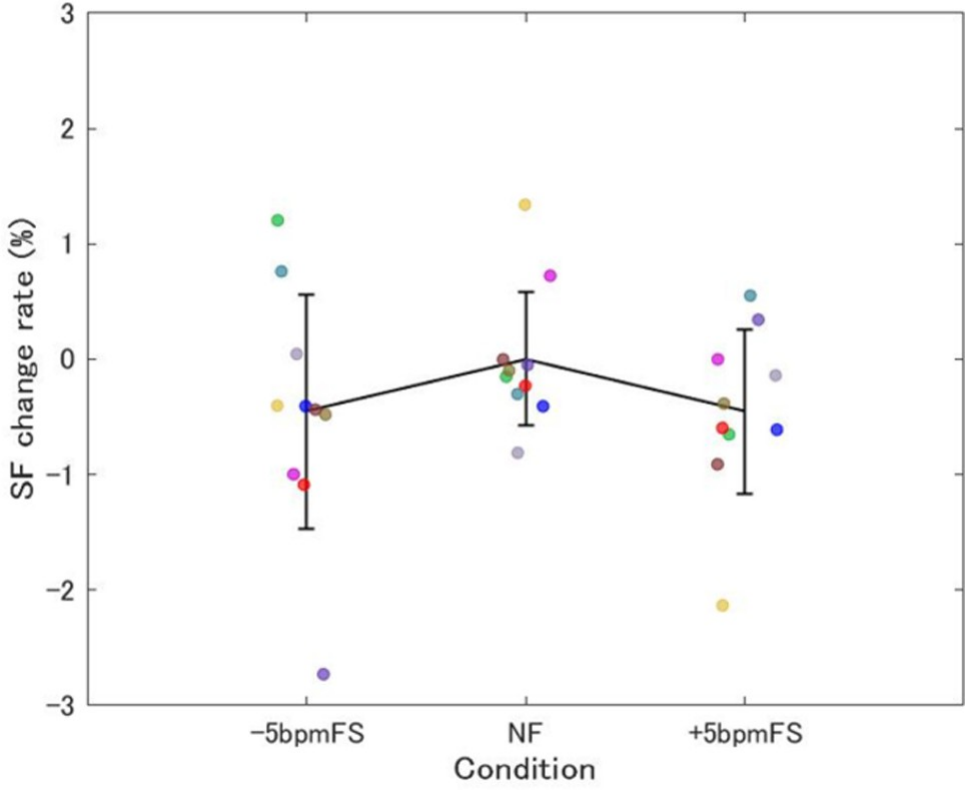
Summarized mean SF change rate in Experiment 1. Each plot shows data for one participant and the data are the mean of the Step frequency (SF) change rate from 5 min after the start of each trial to the end of the trial. The data for the same participants are plotted in the same color. The means for each condition are connected by lines and the error bars represent between-participant SD. The plots were concentrated around 0% in NF, whereas the variability was larger in -5bpmFS and +5bpmFS.

The summarized mean SF change rate from 5 min after the start of each trial to the end is shown in Fig 3. While the NF data were concentrated around 0%, the variation for the ±5bpmFS seemed to be larger.

**Fig 4:**
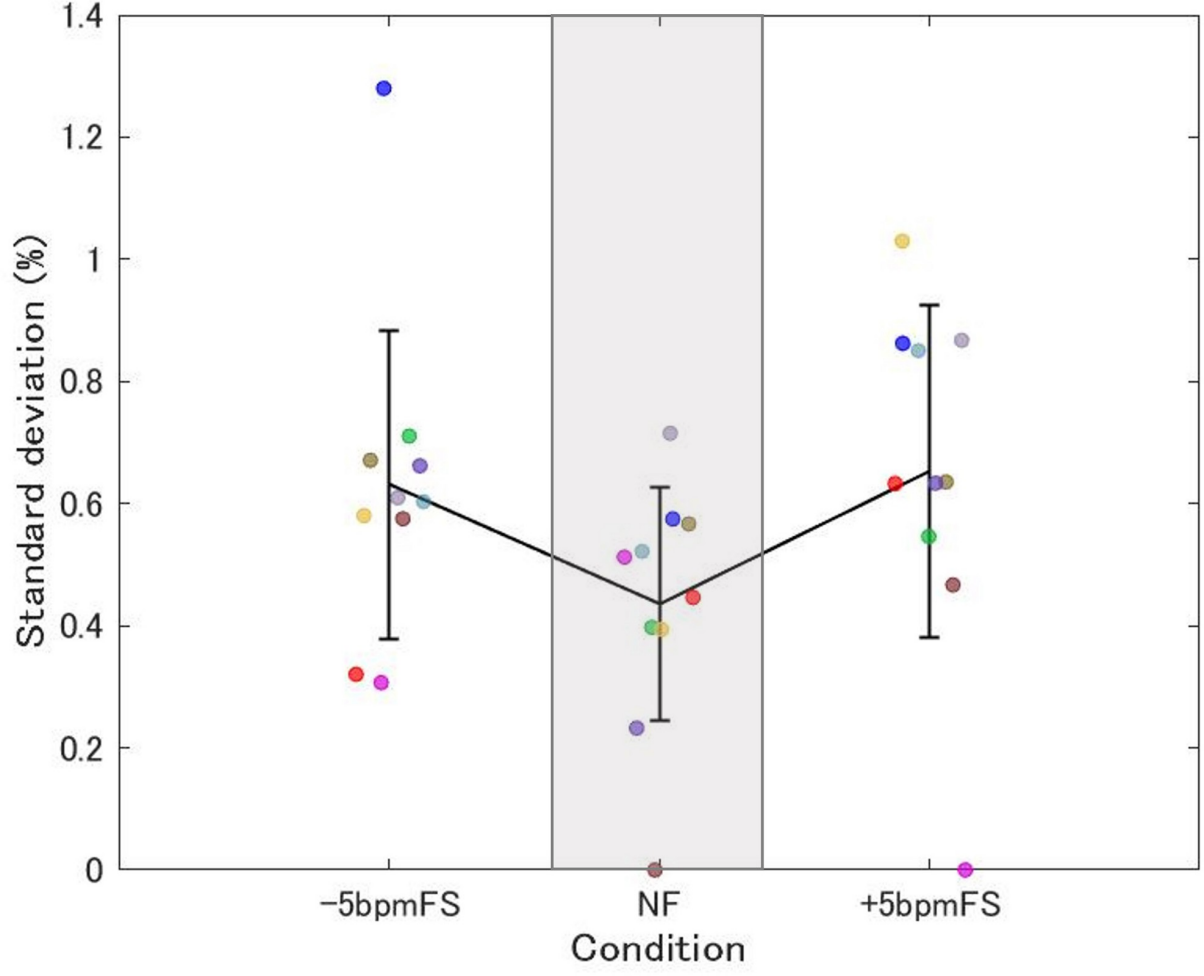
Mean SD of SF change rate in Experiment 1. Each plot shows data for one participant, and the data are the mean SD of the step frequency (SF) change rate from 5 min after the start of each trial to the end. The data for the same participants are plotted using the same color. The means for each condition are connected by lines and the error bars represent between-participant SD.

Fig 4 shows the mean SD of the SF change rate from 5 min after the start of each trial to the end. One-way (condition) ANOVA showed that there was a marginally significant main effect of condition on the SD of SF change rate (F[2, 18] = 3.17, *p* = 0.066, $\eta_{p}^{2}$ = 0.26). Multiple comparisons showed there were no differences between conditions.

### HR

**Fig 5:**
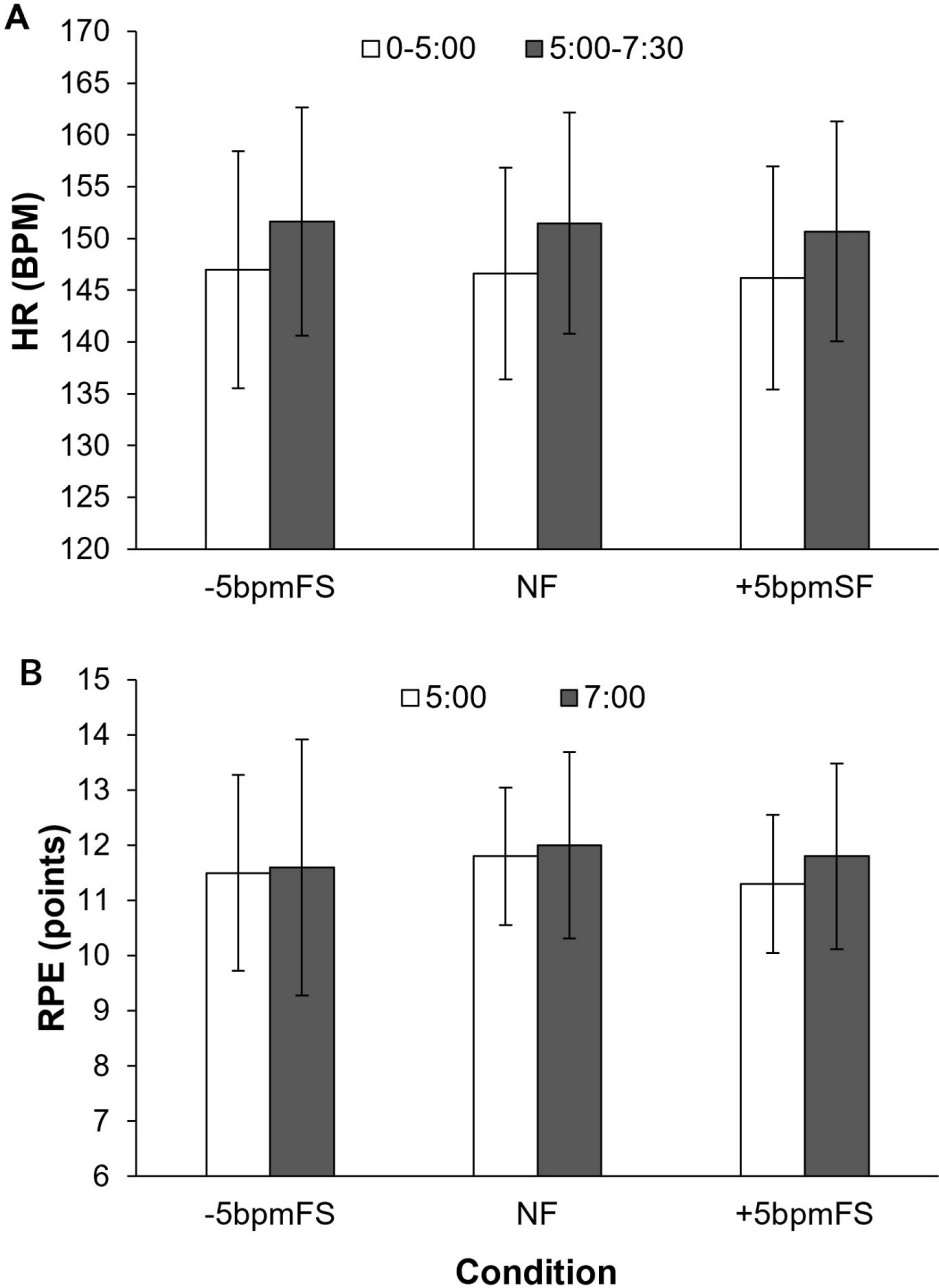
Mean HR and RPE in Experiment 1. HR (A) was averaged from 0 to 5 min and 5 to 7 min and 30 s, and RPE (B) was at 5 and 7 min after the start of each trial in each condition. The error bars represent the between-participant SD. There was no difference in HR or RPE between conditions.

Fig 5A shows the mean HR from 0 to 5 min, and from 5 to 7 min and 30 s in each condition. To investigate the effect of the different footstep tempos of the NR, the HR was divided into two sections: 0 to 5 min, where the NR’s footsteps and the participant’s SF were synchronized; however, were different after 5 min. A two-way (condition × time) ANOVA was performed on the HR, and the results showed that there was no interaction (F[2, 18] = 0.22, *p* = 0.80, $\eta_{p}^{2}$ = 0.024) and no main effect of condition on HR (F[2, 18] = 0.70, *p* = 0.51, $\eta_{p}^{2}$ = 0.072). However, the main effect of time on HR was significant (F[1, 9] = 74.23, *p* = 0.000, $\eta_{p}^{2}$ = 0.89).

### RPE

Fig 5B shows the mean RPE at 5 min and 7 min after the start of each trial. A two-way (condition × time) ANOVA for RPE showed no interaction (F[2, 18] = 1.16, *p* = 0.34, $\eta_{p}^{2}$ = 0.11), and there was no main effect of either condition (F[2, 18] = 0.56, *p* = 0.58, $\eta_{p}^{2}$ = 0.058) or time (F[2, 18] = 1.47, *p* = 0.26, $\eta_{p}^{2}$ = 0.14).

## Discussion

### Effect of the footsteps of the NR on SF

In this study, we examined whether the different footstep tempos of the NR affected the main runners’ SF. The participants ran at a constant speed for 7 min and 30 s, and listened to the footsteps of the NR with the same tempo for the first 5 min, and then to the footsteps of the NR whose tempo was 5 bpm faster or slower after 5 min. The SF of well-trained runners was reported to show small variability when they run at around comfortable speed [31]. In this study, we used a running speed that was considered to be comfortable, and when there was no perceptual information from the NR in the NF condition, the SF change rate showed little variation and the SD was small. However, when the main runner heard the footsteps of the NR and the tempo change (±5bpmFS), the SF change rate was highly variable, and the SD was large. This suggests that the footsteps of the NR may have affected the SF of the main runner.

The study aimed to investigate the effect of the footsteps of the NR on the SF of a main runner’s free-running motion without instruction. For this, the original purpose was not communicated, and the participants were instructed that the experiment investigated the HR when running alone and with others. As an interview was conducted after each running condition, one of the participant said, "I felt that the SF of the person next to me became faster," and recognized the change in tempo of the footsteps of the NR. However, there was no statement that they tried to match SF to the faster footsteps, and there was no major change in the SF change rate. This suggests that the SF fluctuation was not affected by the intention of this study, but occurred spontaneously. Another participant reported that he intentionally tried to match his SF to the footsteps of the NR in the -5 bpm condition. The SF decreased along with the tempo of the footsteps of the NR; however, the decrease of 5 spm did not occur in 5 s along with the change in the tempo, but gradually decreased over approximately 1 min. The participants were not instructed to synchronize their SF with the footsteps of the NR, indicating that the footsteps may encourage intentional and spontaneous SF synchronization. The other participants did not report intentionally trying to match their SF to the tempo of the NR footsteps.

### SF entrainment by the footsteps of the NR

Dyck et al. [16] found that a tempo change of 3% or less from the NR’s SF caused its entrainment in music. In the ±5bpmFS, the footstep tempo was changed by ±5 bpm, which corresponds to a change of approximately ±3% from the NR’s SF.

Similar to music, it was expected that SF would increase with a tempo change of +5 bpm and show a decrease of -5 bpm for footsteps, but no significant differences were found between the conditions. This may have been because of a large change in footstep tempo. The closer the music tempo was to the NR’s SF, the greater the entrainment effect [16]. However, it has been shown that a large frequency difference between two oscillators results in smaller entrainment effects and larger SD of the oscillation frequency [32]. In this study, the increased SD of SF under conditions with NR suggests that the tempo change was beyond the entrainment basin. We conducted Experiment 2 by adding more tempo conditions, with the hypothesis that the entrainment effects would occur with tempo changes closer to the main runners’ SF.

## Results of Experiment 2

### SF change rate

**Fig 6:**
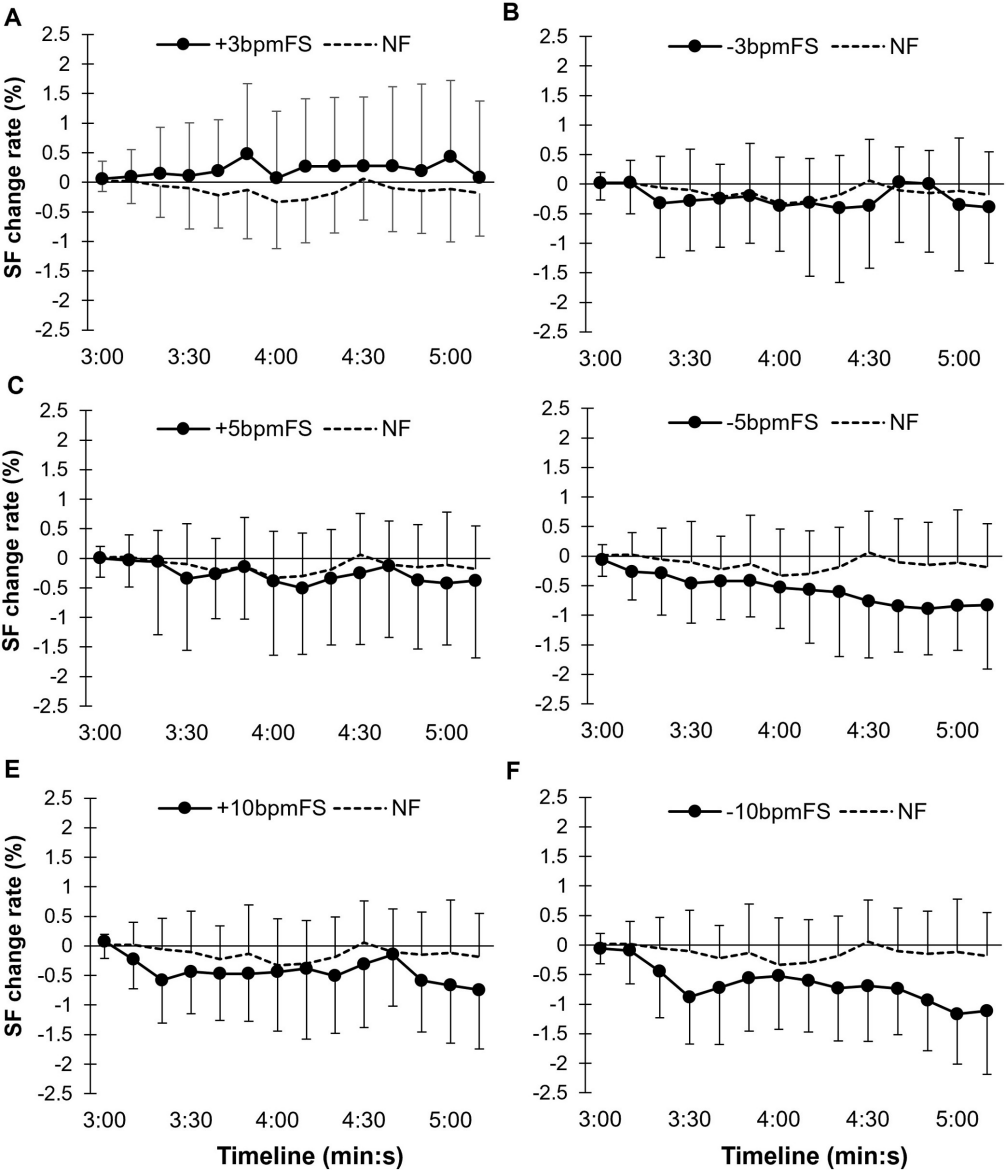
Changes in the SF change rate every 10 s in Experiment 2. The error bars represent between-participant SD. Both +5bpmFS (C) and -5bpmFS (D) showed a tendency to decrease, reproducing the results of Experiment 1. The increase in the SF at +3bpmFS was consistent with the hypothesis. There was a significant difference between the NF and -10bpmFS as well as -10bpmFS and +3bpmFS.

Fig 6 shows the SF change rate every 10 s in the ±3 bpm footsteps condition (±3bpmFS), the NF, the ±5 bpm footsteps condition (±5bpmFS), and the ±10 bpm footsteps condition (±10bpmFS). A two-way ANOVA (condition × time) showed that there was no interaction between condition and time (F[8.21, 114.90] = 1.11, *p* = 0.36, $\eta_{p}^{2}$ = 0.073), with the former having a significant main effect on the SF change rate (F[6, 84] = 3.25, *p* = 0.006, $\eta_{p}^{2}$ = 0.19). As a result of multiple comparisons using the Bonferonni test, there was a significant difference between the NF and -10bpmFS　(*p* = 0.010). Moreover, a significant difference between -10bpmFS and +3bpmFS was found (*p* = 0.011). Time had a significant main effect on the SF change rate (F[3.90, 54.54] = 2.59, *p* = 0.048, $\eta_{p}^{2}$ = 0.16).

**Fig 7:**
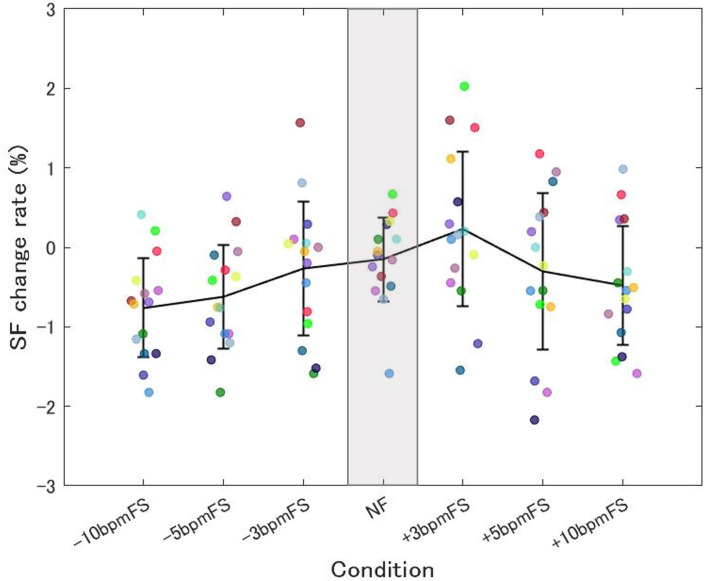
Summarized mean SF change rate in Experiment 2. Each plot shows data for one participant and the data are the mean of the Step frequency (SF) change rate from 3 min after the start of each trial to the end of the trial. The data for the same participants are plotted using the same color. The means for each condition are connected by lines, and the error bars represent between-participant SD. The plots were concentrated around 0% in NF, whereas the variability was larger in the other conditions. The step frequency of the main runner could be close to the footstep tempo of a neighboring runner when it ranged from -10 bpm to +3 bpm.

Fig 7 shows the summarized mean SF change rate from 3 min after the start of each trial to the end of the trial.

**Fig 8:**
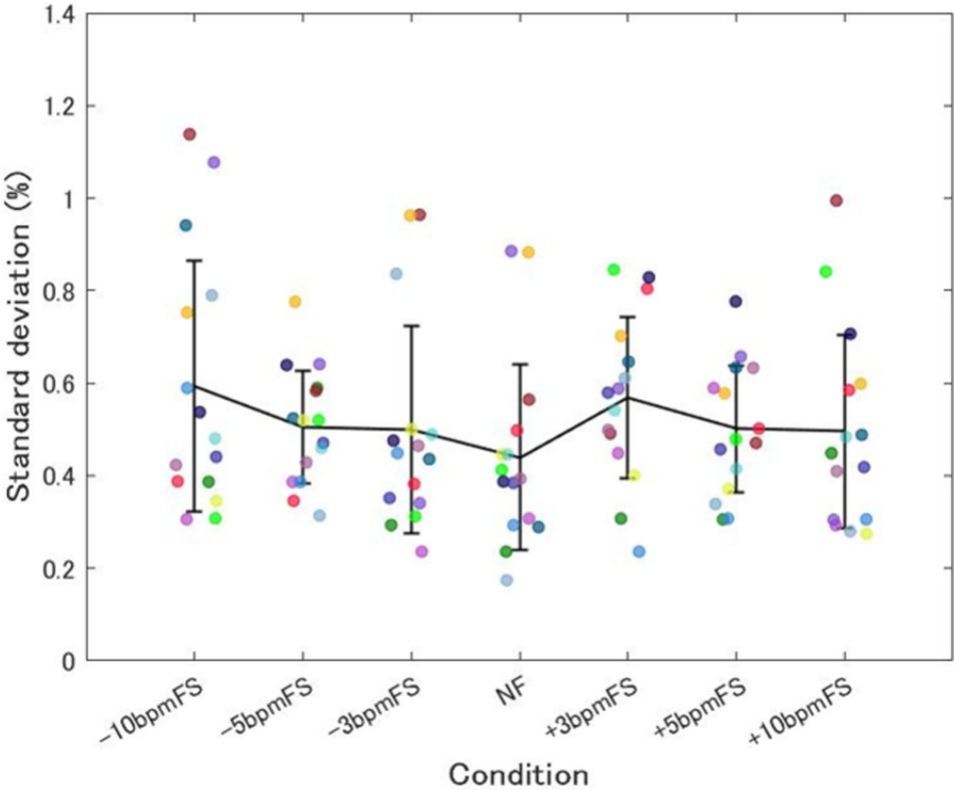
Mean SD of SF change rate in Experiment 2. Each plot shows data for one participant and the data are the mean SD of the step frequency (SF) change rate from 3 min after the start of each trial to the end. The data for the same participants are plotted using the same color. The means for each condition are connected by lines, and the error bars represent between-participant SD. NF showed the lowest mean and replicated Experiment 1, but there were no significant differences among conditions.

Fig 8 shows the mean SD of the SF change rate from 3 min after the start of each trial to the end. One-way (condition) ANOVA showed that there was no significant main effect of condition on the SD of SF change rate (F[6, 84] = 1.32, *p* = 0.26, $\eta_{p}^{2}$ = 0.086).

### HR and RPE

**Fig 9:**
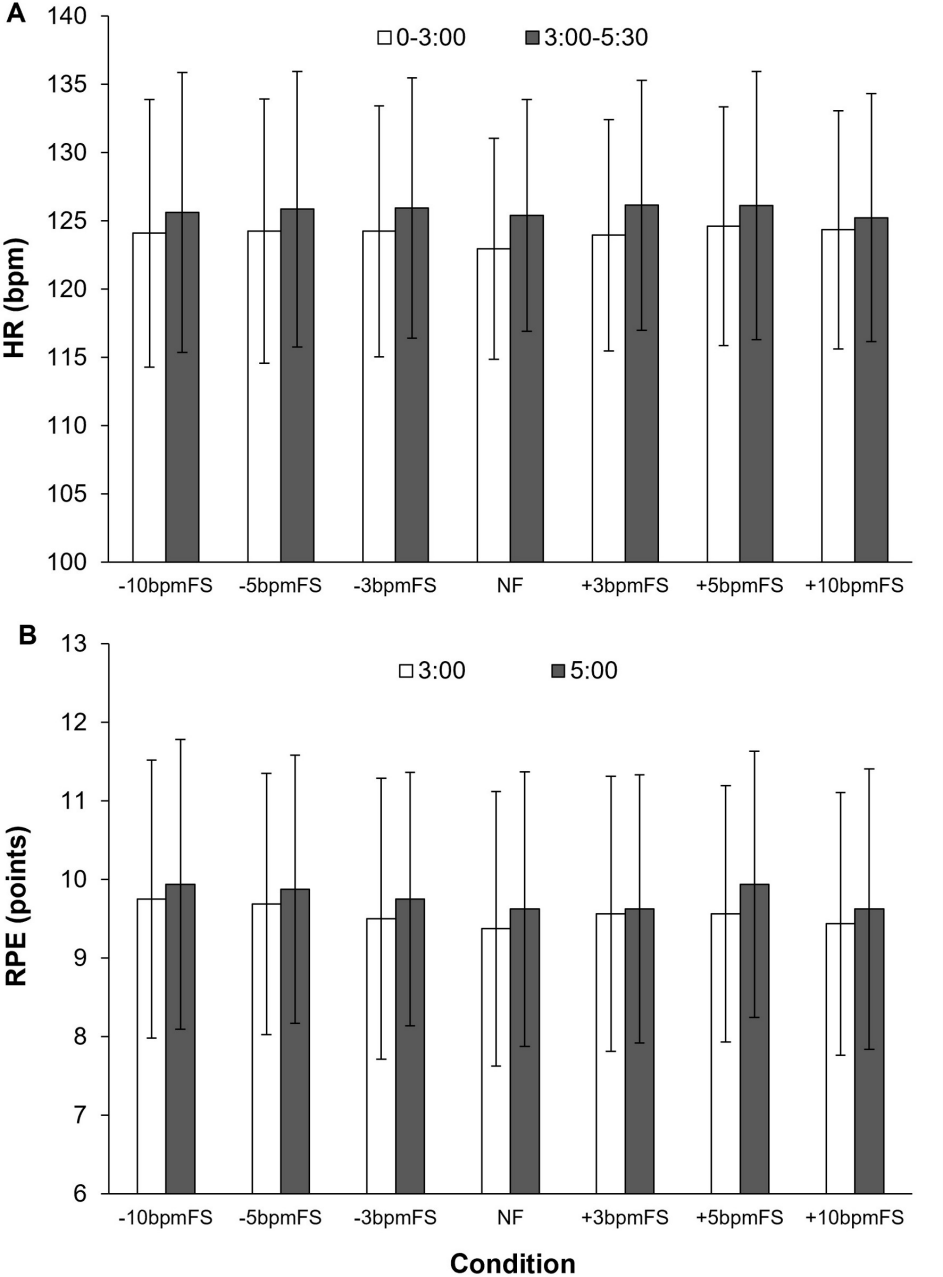
Mean HR and RPE in Experiment 2. HR (A) was averaged from 0 to 3 min and 3 to 5 min and 30 s, and RPE (B) was at 3 and 5 min after the start of each trial in each condition. The error bars represent the SD. There was no difference in HR or RPE between conditions.

Fig 9A shows the mean HR from 0 to 3 min, and from 3 to 5 min and 30 s in each condition. To investigate the effect of the different footstep tempos of the NR, the HR was divided into two sections: 0 to 3 min, where the footsteps of the NR and the participant’s SF were synchronized, and after 3 min, where the footstep tempo of the NR and the participant’s SF were different. As a result of a two-way (condition × time) ANOVA for HR, there was no interaction (F[6, 84] = 0.99, *p* = 0.44, $\eta_{p}^{2}$ = 0.066) and no main effect of condition (F[6, 84] = 0.42, *p* = 0.87, $\eta_{p}^{2}$ = 0.029). Time had a main effect on HR (F[1, 14] = 11.30, *p* = 0.005, $\eta_{p}^{2}$ = 0.45).

Fig 9B shows the RPE at 3 and 5 min after the start of each trial in each condition. A two-way (condition × time) ANOVA for RPE showed there was no interaction (F[6, 84] = 0.33, *p* = 0.92, $\eta_{p}^{2}$ = 0.023) and no main effect of condition (F[6, 84] = 0.49, *p* = 0.82, $\eta_{p}^{2}$ = 0.034). Time had a main effect on RPE (F[1, 14] = 7.98, *p* = 0.014, $\eta_{p}^{2}$ = 0.36).

## Discussion

The participants ran at a comfortable constant running speed for 5 min and 30 s. After hearing the footsteps of a NR whose SF and tempo were the same as theirs for the first 3 min, they ran 3, 5, or 10 bpm faster (+3bpmFS, +5bpmFS, +10bpmFS) or 3, 5, or 10 bpm slower (-3bpmFS, -5bpmFS, +10bpmFS) after 3 min.

### Recognition of research purpose by the participants

In Experiment 2, the participants were not told the original purpose of the study but were informed that it investigated HR when running alone and with others. In the interviews assessing the participants’ impressions after each condition, there was no indication that they were aware that the purpose was to measure SF. This suggests that the participants’ SF fluctuations were not affected by the purpose of the study and occurred spontaneously.

### SF entrainment by the footsteps of the NR

We examined whether the change in footstep tempo closer (±3 bpm) to the main runner’s SF than ±5 bpm or farther away (±10 bpm) caused the participants’ SF to be entrained. As hypothesized, there was a trend toward increased SF at +3 bpmFS, and a significant difference was detected between NF and + -10 bpm as well as +3 bpm and -10 bpm. These results partially support the hypothesis that footstep tempo changes cause SF entrainment.

It has been shown that the SF of two people walking or running side-by-side approached each other [13, 26–28, 33, 34] and it has been confirmed that footsteps are a factor that causes entrainment during walking [26–28]. However, synchronization with external information is less likely to occur at higher exercise intensities [35], and it may be less likely while running, where exercise intensity is higher than in walking.

### SF is difficult to increase and easy to decrease

Overall, compared to NF, the SF seemed to decrease under all conditions except +3 bpmFS. Previous studies reported that the ratio of SF increase to SF decrease is small and the ratio of SF increase to SF decrease is large owing to the entrainment of music [16, 17, 36]. The same asymmetric trend was observed in the present study.

## General discussion

This study aimed to investigate the effects of footsteps of a NR on the main runner’s SF, HR, and RPE. In Experiment 1, the participants ran at a comfortable constant speed for 7 min and 30 s, and after listening to the footsteps of a NR with the same tempo for the first 5 min, and then to the footsteps of a NR whose tempo was 5 bpm faster (+5bpmFS) or slower (-5bpmFS) after 5 min. In Experiment 2, the wider range of tempo conditions were adopted than those in Experiment 1; the participants heard footsteps 3 bpm, 5 bpm, and 10 bpm faster (+3bpmFS, +5bpmFS, and +10bpmFS) or 3 bpm, 5 bpm, and 10 bpm slower (-3bpmFS, -5bpmFS, and -10bpmFS).

### Different tempo changes of the footsteps of the NR affect the main runner’s SF

In Experiment 1, the effect of NR footsteps on the SD of SF was observed. Experiment 2, wherein the footstep tempo condition was added, showed the effect of NR footsteps on SF. In both Experiments 1 and 2, there was a decreasing trend in SF at ±5 bpmFS compared to NF, although it was not statistically significant. The main effect of time on the SF was also consistent between Experiments 1 and 2. These indicate the reproducibility of the results. These results showed that a change in the footstep tempo of NR caused a different SF fluctuation in the main runner than in its absence. It has been shown that auditory stimuli with a certain periodicity can activate several brain structures including the basal ganglia, which is considered to be a brain region that modulates locomotion, and that predicting the tempo of auditory stimuli can promote the activation [37]. Several studies have shown that auditory stimuli such as music with a periodic tempo and metronomes affect the SF of runners [16, 17, 20]. These auditory stimuli can easily predict the timing of beat to beat, resulting in significant activation of the basal ganglia, which is thought to affect the tempo of the movement (SF).

### Auditory-motor synchronization

The tempo of a movement is entrained into a specific cycle or phase in response to external sensory stimuli. This is also called sensorimotor synchronization [38]. Specifically, the synchronization of the motor tempo with rhythmic auditory stimuli is called auditory-motor synchronization. Auditory-motor synchronization studies to date have included both instructed and uninstructed synchronization experimental paradigms, although most previous studies on auditory-motor synchronization have focused on instructed synchronization tasks, such as handheld pendulum swinging [39, 40], dancing [41, 42], and tapping [15, 43].

However, auditory-motor synchronization can occur spontaneously even when the participant is not instructed to match the tempo of the movement to the auditory stimulus [16, 44]. For example, synchronization between footstep sound and steps has been extensively studied in walking. Nessler et al. [26] conducted an experiment in which two participants walked on two adjacent treadmills, and their visual or auditory information were blocked, or they also walked hand in hand. In each of these conditions, synchronization between the two walkers occurred without any instruction, indicating that it can be spontaneous between two people walking side-by-side if they are provided with visual, auditory, or tactile sensory information of the other person.

Although these results partially supported the hypothesis that footstep tempo changes cause SF entrainment, there were no significant differences except between NF and -10 bpm, which is not sufficient to adequately support the hypothesis. This may be due to an increased internal focus of attention caused by a higher intensity of exercise compared to walking. As exercise intensity increases, physiological sensations dominate the attention and focus on external bodily information is reduced [45, 46]. Reduced allocation of attention to the partner causes less interpersonal synchrony [47, 48]. Many participants reported their feelings about muscle status and running movements, and this increased internal focus of attention might have interfered with synchrony. Further empirical research is needed, including experiments with different exercise intensities.

### HR and RPE

In Experiments 1 and 2, the effects of NR footsteps on the main runners’ HR and RPE were examined. No differences were found among the conditions. In previous studies, synchronous music and synchronous metronomes have been shown to affect HR, oxygen uptake, and performance [22, 23, 49, 50]. Although music has been shown to affect RPE [21], effects of beat sounds without melody or harmony (e.g., metronome) have not been observed. Since the footsteps of other runners are a beat sound with a constant tempo without melody or harmony, they do not reduce the psychological load during running but improve the physiological load. In the present study, the RPE was not changed by listening to the footsteps of the NR, and the HR did not change. It has been shown that each runner has a unique optimal SF that minimizes physiological load [14]. Therefore, it is possible that the change in the tempo of the footsteps of the NR did not lead to an improvement in the physiological load because the NR deviated from the unique optimal SF.

In the present study, considering the effect of fatigue on SF, the exercise intensity was set within the range of running speed and total running time, which were reported not to cause fatigue in previous studies [16]. It is possible that the relatively low intensity of exercise did not affect the physiological load due to footsteps. Future studies should examine the effects of other runners’ footsteps on physiological effects and performance at exercise intensities above the anaerobic work threshold and at running speeds closer to race conditions.

### Limitations

This study has some limitations. Previous studies reported a relationship between the number of attentional resources directed toward a partner and the occurrence of interpersonal synchronization during side-by-side walking [48]. However, the allocation of attentional resources in a laboratory setting was different (e.g., attention tended to be directed to footsteps or vice versa), and the results may differ from those in over-ground settings obtained through over-ground running.

In this study, the exercise intensity was low to moderate. Hence, it is unclear how important footsteps were for physiological and psychological load and performance in high-intensity races. Future research is required to examine and understand these issues.

## Conclusion

We examined the effect of the footstep sounds of adjacent runners on the SF of trained runners. The results showed that the footstep sounds of adjacent runners can partially influence the mean and variability of step frequency, suggesting that running step characteristics can be unintentionally modulated by auditory information generated by others during running. Future research should examine the effects of multimodal information in a wide field environment, such as actual long-distance running competitions.

## Supporting information

S1 Table Individual running speed in Experiments 1 and 2. (PDF)
