# Supplementary material for: Auditory interaction between runners: Does footstep sound affect step frequency of neighboring runners?
Source: PLoS One. 2023 Jan 6;18(1):e0280147. doi: 10.1371/journal.pone.0280147 (PMC9821460; doi:10.1371/journal.pone.0280147)
Supplement: S1 Table — (PDF) [file pone.0280147.s001.pdf]

**Experiment 1**		**Experiment 2**	
**#Participant**	**Running speed (km/h)**	**#Participant**	**Running speed (km/h)**
**1**	13.3	**1**	12.0
**2**	12.9	**2**	12.9
**3**	12.9	**3**	12.0
**4**	13.5	**4**	13.0
**5**	12.7	**11**	13.3
**6**	12.6	**12**	12.0
**7**	11.8	**13**	10.5
**8**	12.5	**14**	12.5
**9**	13.5	**15**	13.0
**10**	11.8	**16**	12.9
		**17**	13.0
		**18**	12.0
		**19**	12.0
		**20**	11.0
		**21**	12.0
**Mean**	12.75	**Mean**	12.27

Note. A total of four participants (#1-4) participated in Experiments 1 and 2.
